# Supplementary material for: Altered somatic hypermutation patterns in COVID-19 patients classifies disease severity
Source: Front Immunol. 2023 Apr 19;14:1031914. doi: 10.3389/fimmu.2023.1031914 (PMC10154551; doi:10.3389/fimmu.2023.1031914)
Supplement: Supplementary file 1 [file DataSheet_1.pdf]

Altered somatic hypermutation patterns in COVID-19 patients classifies disease severity

Modi Safra[1,2], Zvika Tamari[1,2], Shachaf Shiber[3,4], Avi Peretz[5,6], Moshe Matan[5], Eli Ben-Chetrit[7], Hani Karameh[8], Yigal Helviz[9], Pazit Polak[1,2], Baruch Brenner[4,10], Adva Levy-Barda[11], Vered Yahalom[4,12], Tamir Tuller[13], Meital Gal-Tanamy[6], and Gur Yaari[1,2,*]

[1]Bio-engineering, Faculty of Engineering, Bar Ilan University, Ramat Gan, Israel
[2]Bar Ilan Institute of Nanotechnologies and Advanced Materials, Bar Ilan University, Ramat Gan, Israel
[3]Emergency Department, Rabin Medical Center- Belinson campus, Petah Tikva, Israel
[4]Sackler Faculty of Medicine, Tel Aviv University, Tel Aviv, Israel
[5]Clinical Microbiology Laboratory, Baruch Padeh Medical Center, Poriya, Israel
[6]The Azrieli Faculty of Medicine, Bar-Ilan University, Safed, Israel
[7]Infectious Diseases Unit, Shaare Zedek Medical Center, Hebrew University School of Medicine, Jerusalem, Israel
[8]Jesselson Integrated Heart Center, Shaare Zedek Medical Center, Hebrew University School of Medicine, Jerusalem, Israel
[9]Intensive Care Unit, Shaare Zedek Medical Center, Hebrew University School of Medicine, Jerusalem, Israel
[10]Institute of Oncology, Rabin Medical Center- Belinson campus, Petah Tikva, Israel
[11]Biobank, Department of pathology, Rabin Medical Center- Belinson campus, Petah Tikva, Israel
[12]Blood Services & Apheresis Institute Director, Rabin Medical Center- Belinson campus, Petah Tikva, Israel
[13]Department of Biomedical Engineering and The Sagol School of Neuroscience, Tel Aviv University, Tel Aviv, Israel
[*]Correspondence: Gur Yaari, gur.yaari@biu.ac.il

April 13, 2023

Table S1: **Summary of volunteers' data (bulk sequencing).** All samples were collected in 2020.

Hospital	Age	Sex	Condition	Diagnosis date	Sample date	Sample #
Rabin	NA	NA	mild	NA	23.7	HSCov1
Rabin	23	F	mild	30.7	2.8	HSCov2
Rabin	58	M	mild	1.8	5.8	HSCov4
Rabin	45	M	mild	21.7	9.8	HSCov5
Rabin	55	M	mild	13.8	18.8	HSCov6
Rabin	NA	M	severe	14.8	18.8	HSCov7
Shaare Zedek	39	M	mild	22.4	23.4	HSCov13
Shaare Zedek	31	F	mild	25.4	27.4	HSCov14
Shaare Zedek	44	F	mild	28.4	30.4	HSCov15
Shaare Zedek	43	F	mild	1.5	3.5	HSCov16
Shaare Zedek	30	F	mild	15.5	17.5	HSCov18
Shaare Zedek	59	F	mild	27.5	3.6	HSCov19
Rabin	58	M	mild	26.8	26.8	HSCov20
Rabin	68	M	mild	1.9	1.9	HSCov21
Rabin	75	M	severe	30.8	1.9	HSCov22
Rabin	61	F	mild	20.9	23.9	HSCov24
Rabin	78	M	mild	17.9	24.9	HSCov25
Rabin	61	M	mild	20.9	24.9	HSCov26
Rabin	69	F	mild	23.9	30.9	HSCov27
Rabin	49	M	severe	20.9	30.9	HSCov28
Rabin	52	M	severe	27.9	1.10	HSCov29
Rabin	57	M	mild	29.9	6.10	HSCov30
Rabin	62	F	severe	4.10	6.10	HSCov31
Rabin	86	M	severe	2.10	6.10	HSCov32
Rabin	65	M	mild	29.9	7.10	HSCov33
Rabin	69	M	severe	7.10	7.10	HSCov34
Rabin	42	F	mild	14.10	21.10	HSCov38
Rabin	18	M	mild	1.10	21.10	HSCov39
Rabin	19	F	mild	3.10	21.10	HSCov40
poria	53	F	severe	31.08	6.10	HSCov43
poria	55	F	mild	30.08	4.10	HSCov45
poria	49	F	mild	31.3	4.10	HSCov49
poria	28	M	mild	19.9	4.10	HSCov50
poria	47	F	mild	23.8	4.10	HSCov51
poria	43	M	severe	4.10	6.10	HSCov53
poria	48	F	mild	30.4	4.10	HSCov56
poria	61	F	mild	28.3	6.10	HSCov57
poria	26	F	mild	14.8	4.10	HSCov58
poria	37	M	mild	30.7	4.10	HSCov59
poria	51	M	mild	28.9	6.10	HSCov60

Continued on next page

Table S1 – continued from previous page

Hospital	Age	Sex	Condition	Diagnosis date	Sample date	Sample #
poria	61	M	mild	25.9	6.10	HSCov61
poria	34	M	severe	22.9	6.10	HSCov62
poria	61	M	severe	5.10	6.10	HSCov63
poria	58	F	mild	23.9	8.10	HSCov64
poria	36	M	mild	24.9	8.10	HSCov65
poria	27	M	mild	16.9	8.10	HSCov66
poria	28	F	mild	2.9	8.10	HSCov67
poria	55	F	mild	27.9	6.10	HSCov68
poria	85	M	mild	21.9	6.10	HSCov69
poria	34	M	mild	24.9	8.10	HSCov70
poria	72	M	severe	2.11	3.11	HSCov71

Table S2: **Summary of volunteers' data (single cell sequencing).** All samples were collected at Rabin Hospital, with a mild condition.

Age	Sex	Diagnosis date	Sample date	Sample #
31	F	27.12.20	30.12.20	HSCov73
41	F	28.12.20	30.12.20	HSCov75
39	F	29.12.20	24.1.21	HSCov77
44	M	7.1.21	24.1.21	HSCov78
26	M	23.1.21	24.1.21	HSCov79
22	F	14.10.21	24.1.21	HSCov81
45	M	10.10.21	31.1.21	HSCov83
39	F	15.1.21	31.1.21	HSCov85
58	F	14.1.21	31.1.21	HSCov86
31	F	1.2.21	14.2.21	HSCov87
43	F	3.2.21	14.2.21	HSCov88
28	F	1.2.21	14.2.21	HSCov89
49	M	14.1.21	14.2.21	HSCov90

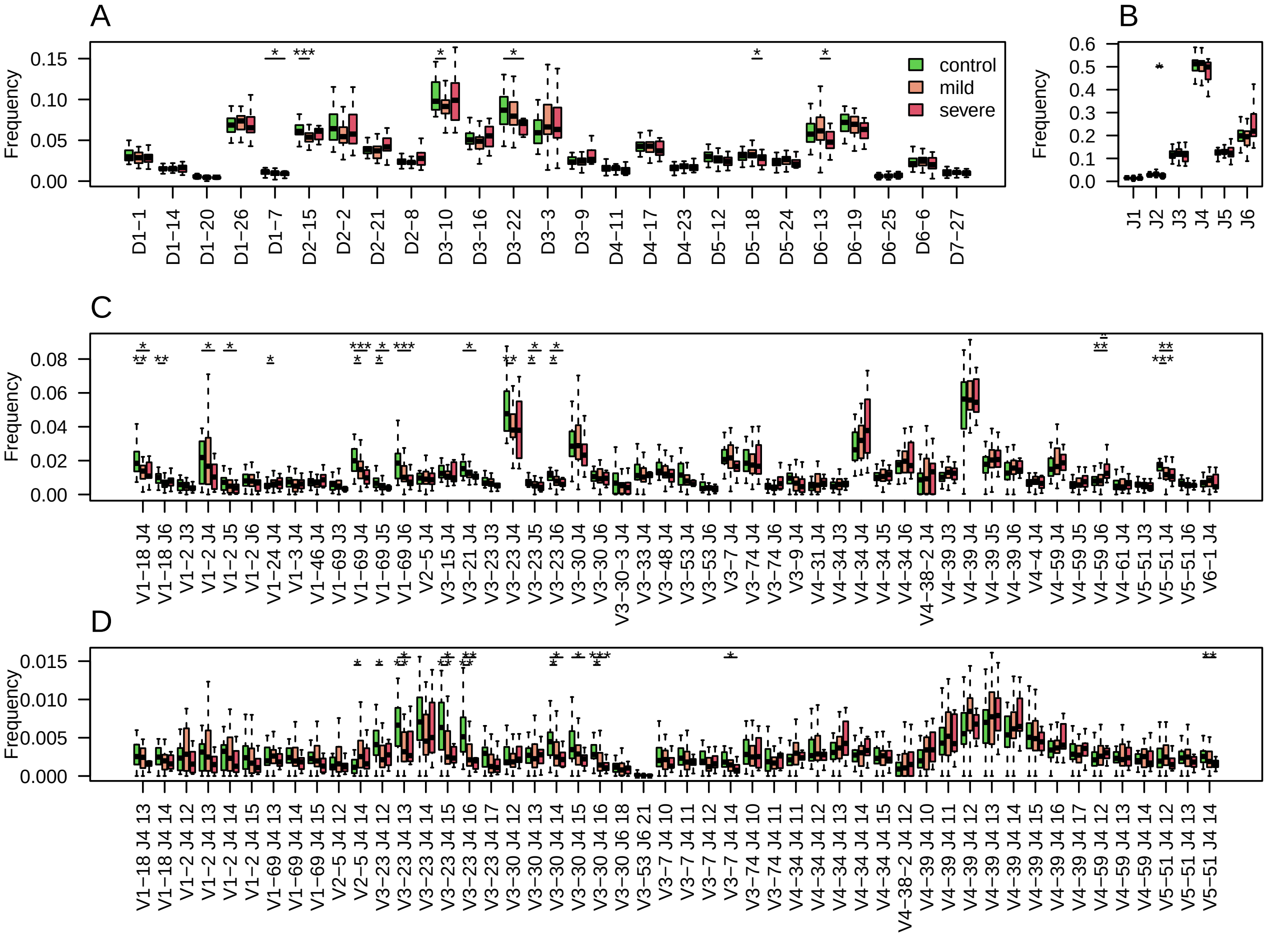

Figure S1: **Characteristics of the heavy chain sequencing data**
A. D gene usage comparison between individuals with COVID-19 at indicated severity levels and healthy controls. B. J gene usage comparison. C. Combinations of V & J gene usage comparison. Shown are the top 50 highest frequencies. D. Clusters comparison between individuals with COVID-19 at indicated severity levels and healthy controls. Shown are the top 50 highest frequencies. Throughout the figure, * marks a P value lower than 0.05, ** marks a P value lower than 0.01, and *** marks a P value lower than 0.001.

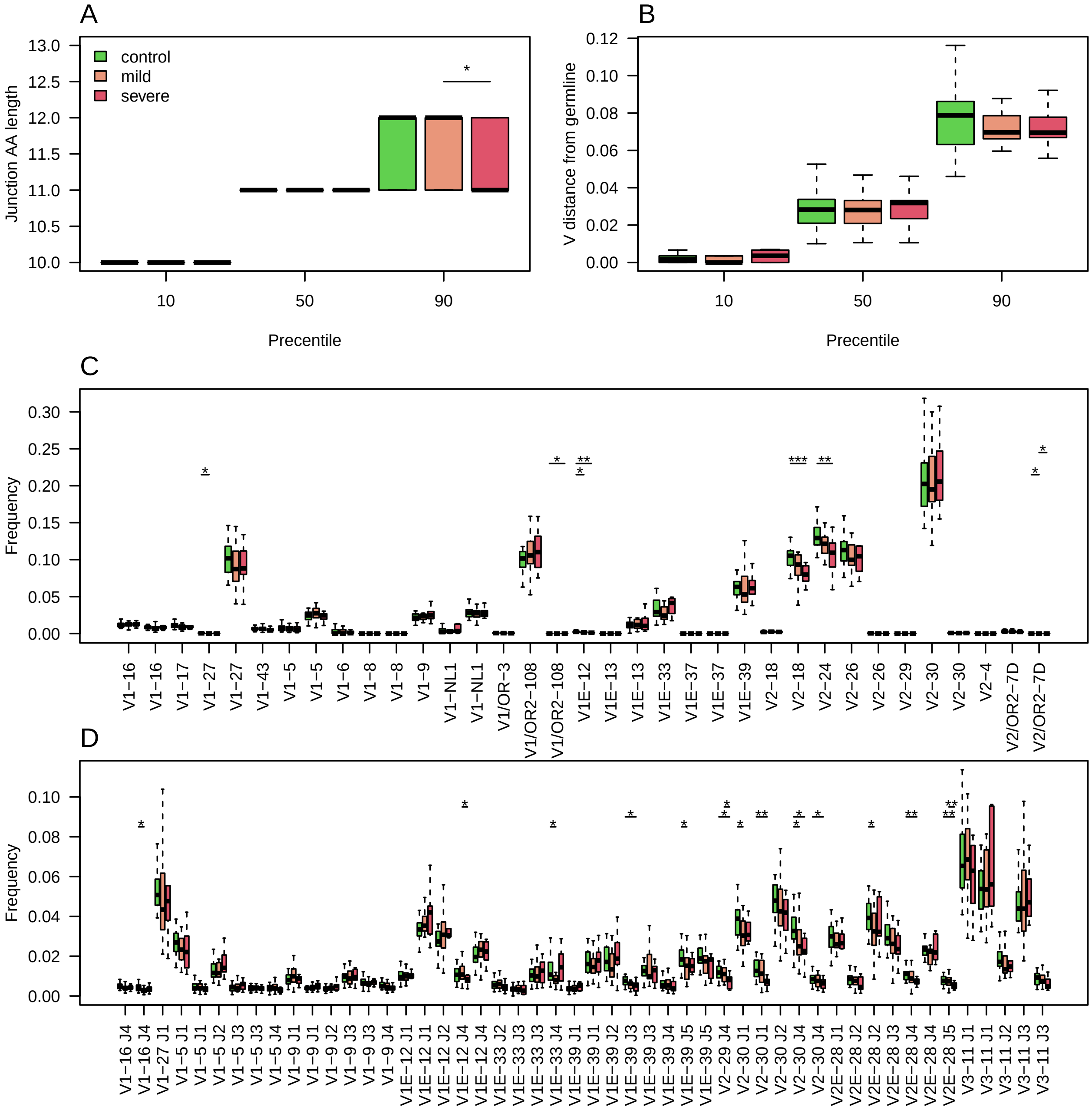

Figure S2: **Characteristics of the light kappa chain sequencing data**
A. 10,50 and 90 percentiles of AA CDR3 lengths in individuals with COVID-19 at indicated severity levels and healthy controls. B. 10,50 and 90 percentiles of V gene distances from germline in individuals with COVID-19 at indicated severity levels and healthy controls. C. Boxplots showing V gene usage in individuals with COVID-19 at indicated severity levels and healthy controls. Shown are the top 50 highest mean frequencies. D. V & J gene usage comparison. Throughout the figure, * marks a P value lower than 0.05, ** marks a P value lower than 0.01, and *** marks a P value lower than 0.001.

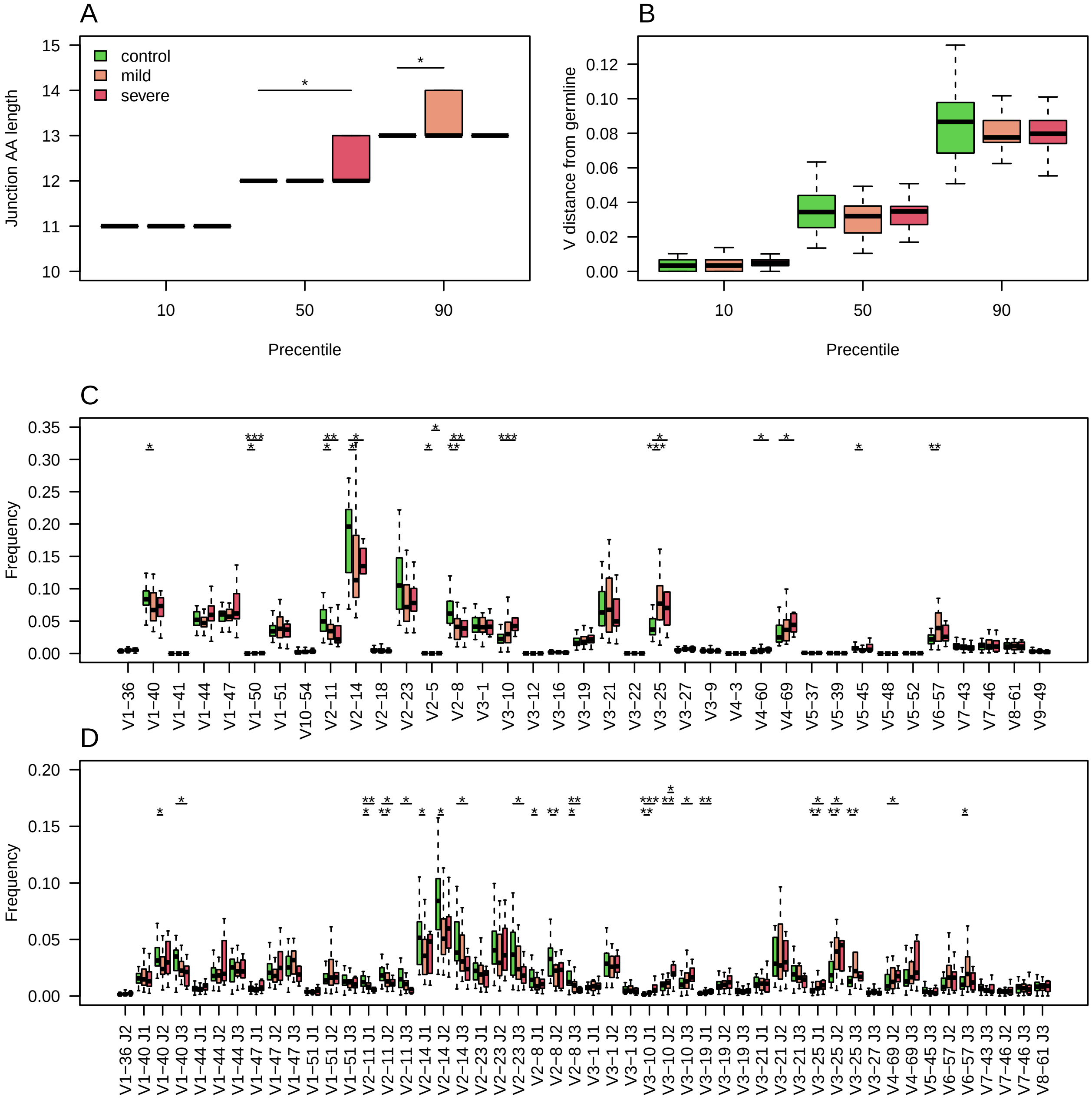

Figure S3: **Characteristics of the light lambda chain sequencing data**
A. 10,50 and 90 percentiles of AA CDR3 lengths in individuals with COVID-19 at indicated severity levels and healthy controls. B. 10,50 and 90 percentiles of V gene distances from germline. C. Boxplots showing V gene usage. Shown are the top 50 highest mean frequencies. D. V & J gene usage comparison. Shown are the top 50 highest mean frequencies. Throughout the figure, * marks a P value lower than 0.05, ** marks a P value lower than 0.01, and *** marks a P value lower than 0.001.

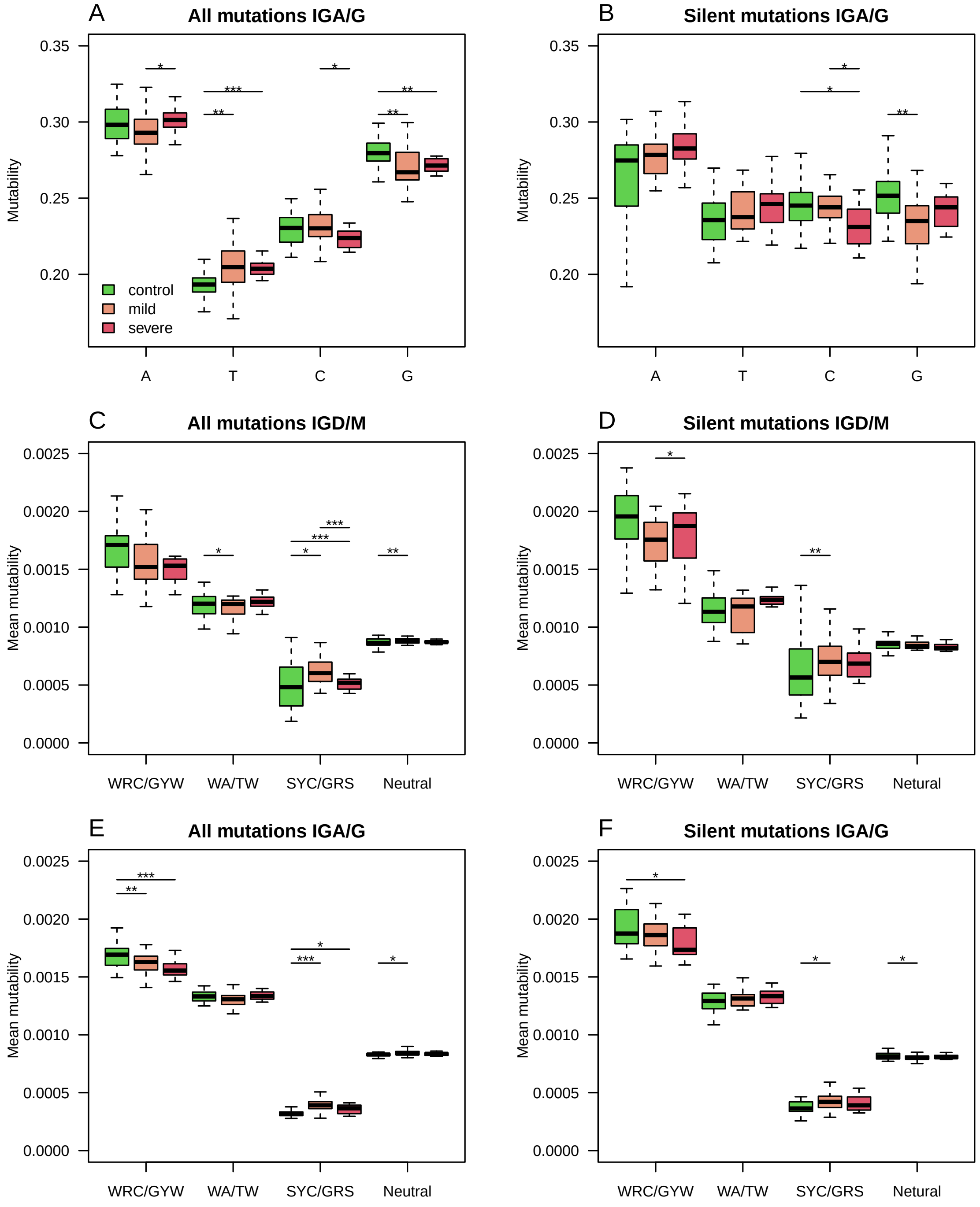

Figure S4: **Silent and replacement mutability in SHM: single base mutability, 5-mers hot-spots and cold-spots**
A. A single base mutability model was built based on IGA/G isotypes of COVID-19 patients and controls, taking into account only one representative from each clone. Shown are boxplots representing the normalized sum of single base mutability. B. The same plot as in A, but for silent mutations only. C-D. A 5-mer SHM model based on both silent and replacement mutations in C, or silent only mutations in D, was built using the IGD and IGM isotypes of COVID-19 patients at different severity levels and healthy controls. Shown are the known SHM hot-spots, SHM cold-spots, and the rest of the sites. E-F. A 5-mer SHM model based on both silent and replacement mutations in E, or silent only mutations in F, was built using the IGA and IGG isotypes of COVID-19 patients at different severity levels and healthy controls. Shown are the known SHM hot-spots, SHM cold-spots, and the rest of the sites. Throughout the figure, * marks a P value lower than 0.05, ** marks a P value lower than 0.01, and *** marks a P value lower than 0.001.

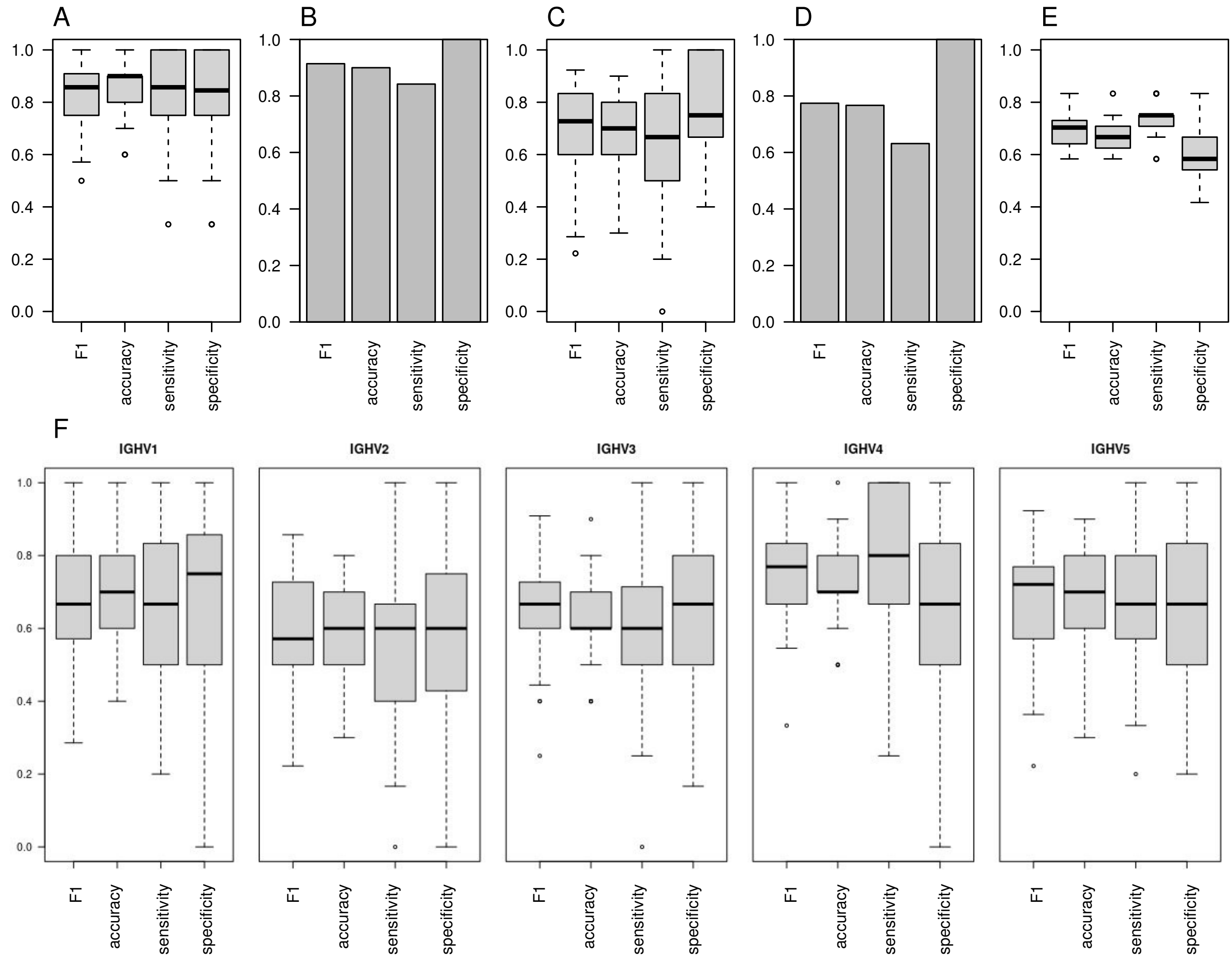

Figure S5: **SHM in the heavy chain enables both COVID-19 classification and severity classification using one representative from each clone, but to less efficiency when building the matrix based on a single V family**

A. An ML algorithm was trained on the substitutions matrix of the 5-mer SHM model (taking into account only one representative from each clone), which was created for the IGA/G isotypes. Boxplots representing F1 score, accuracy, specificity, and sensitivity of 50 random splits to train and test groups are shown. B. Logistic regression was trained on the substitutions matrix of the SHM model built from the entire dataset. Barplot representing F1 score, accuracy, specificity, and sensitivity of classifications on the test group. C. The same algorithm as in A was trained on silent mutations only. Shown are Boxplots representing the F1 score, accuracy, specificity, and sensitivity of 50 random splits to train and test groups. D. The same algorithm as in C was trained on silent mutations only. Shown are barplots representing the F1 score, accuracy, specificity, and sensitivity of classifications on the test group. E. Boxplots showing F1 score, accuracy, specificity, and sensitivity of 20 leave-one-out cross validation of severity classification. Each leave-one-out was on 12 severe COVID-19 patients and 12 randomly selected mild COVID-19 patients. The ML algorithm was trained on the mutability matrix of the SHM cold-spots in these groups. F. F1 score, accuracy, specificity, and sensitivity of classifications based on single V family SHM matrices.

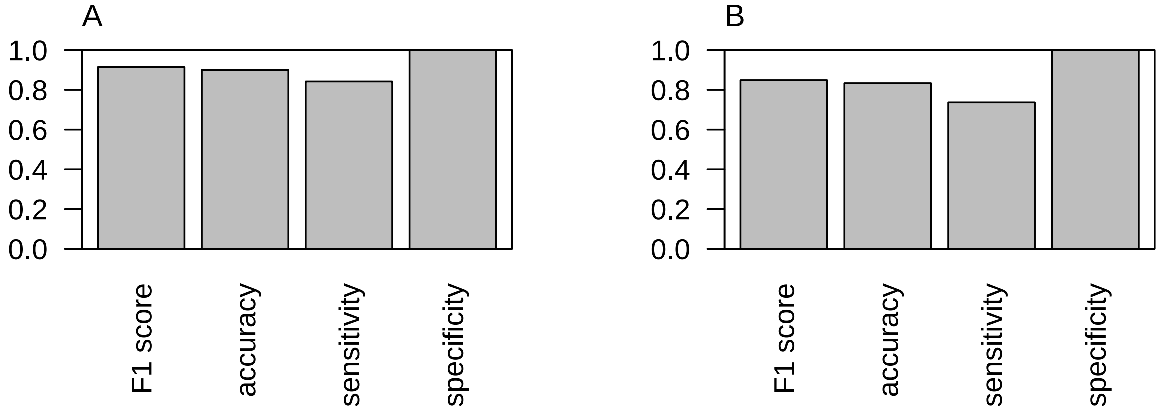

Figure S6: **SHM of heavy chains enables COVID-19 classification - test group**
A. Logistic regression was trained on the substitutions matrix of SHM model built from the entire dataset. A barplot representing F1 score, accuracy, specificity, and sensitivity of classifications on the test group. B. The same algorithm as in A was trained on silent mutations only. Shown are barplots representing the F1 score, accuracy, specificity, and sensitivity of classifications on the test group.

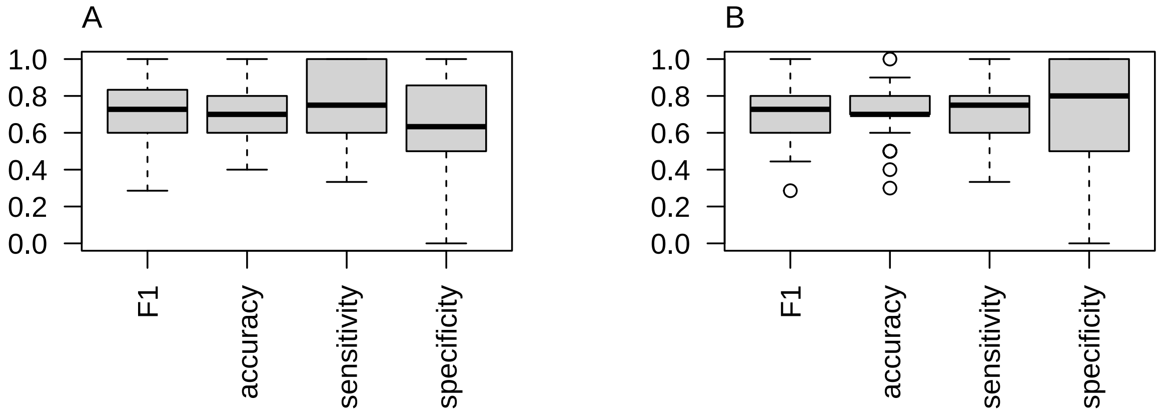

Figure S7: **SHM of light chains enables COVID-19 classification**
A. Logistic regression was trained on the substitutions of SHM model built from the entire dataset. Boxplot representing F1 score, accuracy, specificity, and sensitivity of 50 random splits to train and test groups are shown. B. The same algorithm as in A was trained on silent mutation only. Shown are boxplots representing the F1 score, accuracy, specificity, and sensitivity of 50 random splits to train and validation groups.

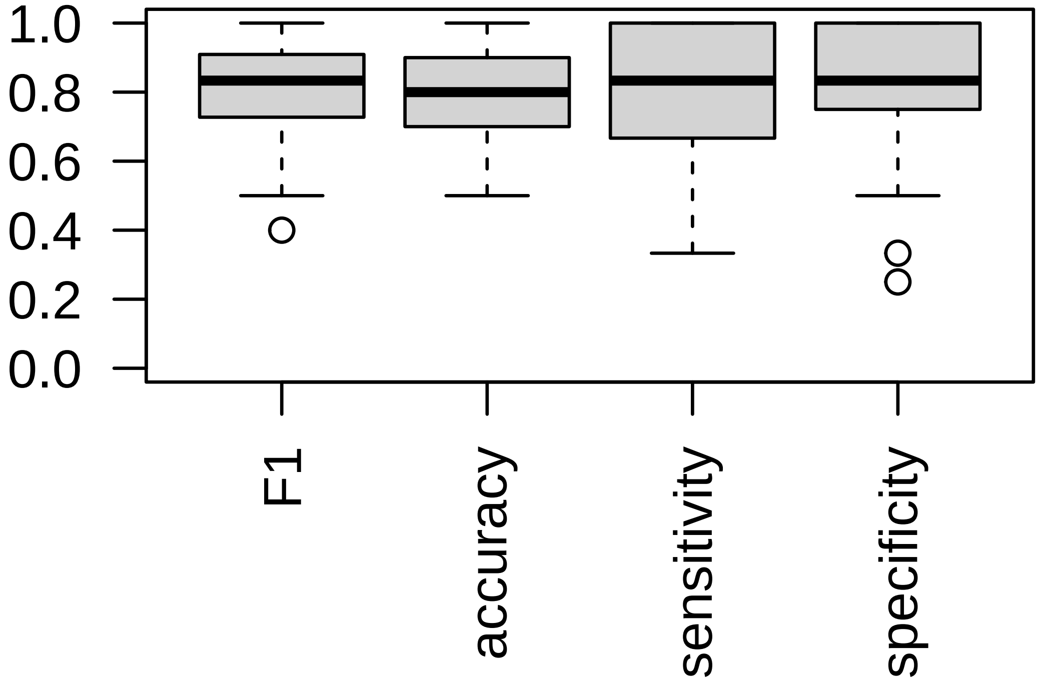

Figure S8: **SHM both Heavy and Light chains enables COVID-19 classification** A. Logistic regression was train on the substitutions of SHM model built on data. A boxplot representing F1 score, accuracy, specificity, and sensitivity of classifications of 50 random splits to train and validation groups are shown.

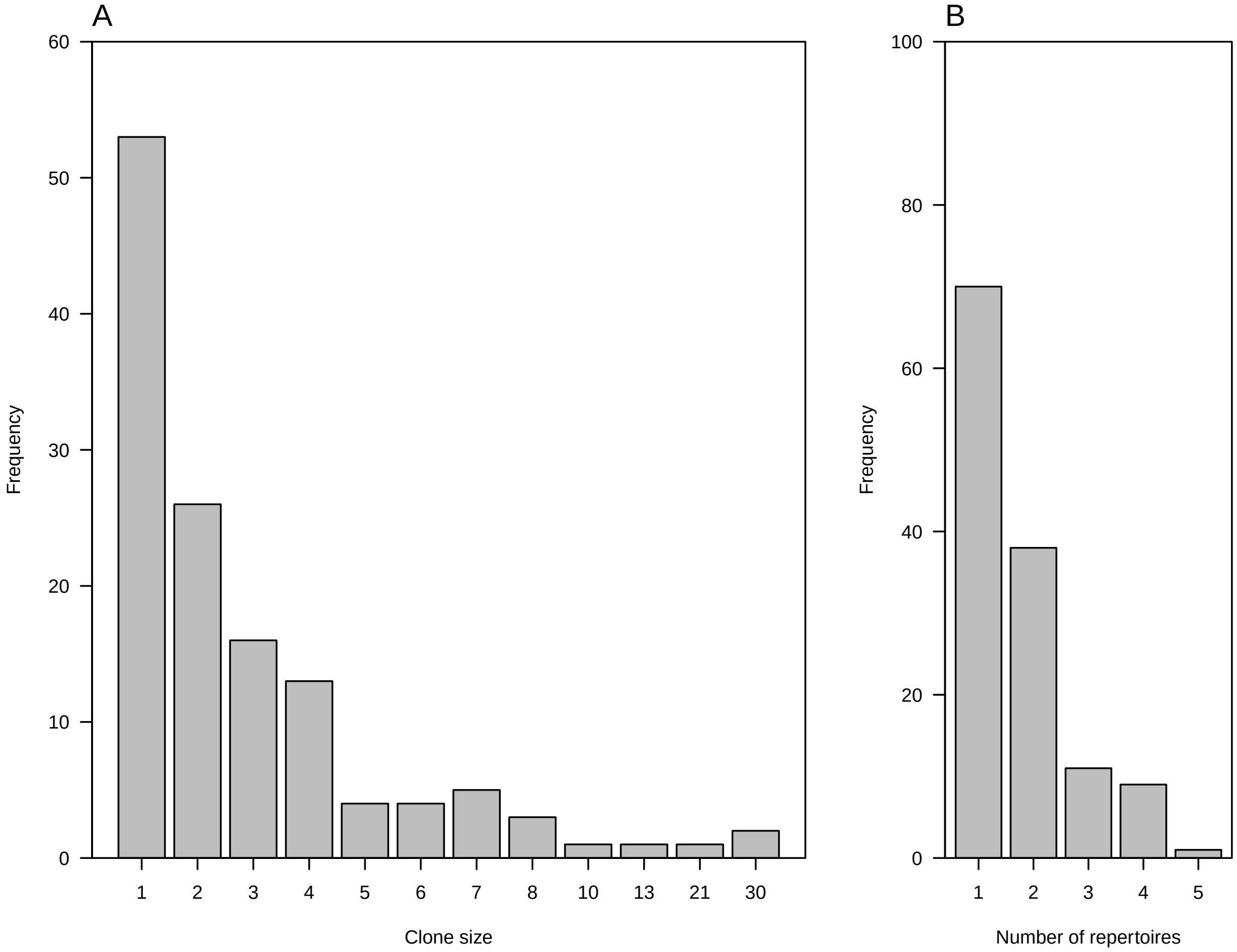

Figure S9: **Characterization of known clones of COVID-19 antibodies**
A. The frequencies of clones found in our COVID-19 patients with indicated clones sizes. B. The frequencies of clones found in our COVID-19 patients with the indicated number of repertoires having at least one sequence which belongs to the clone.

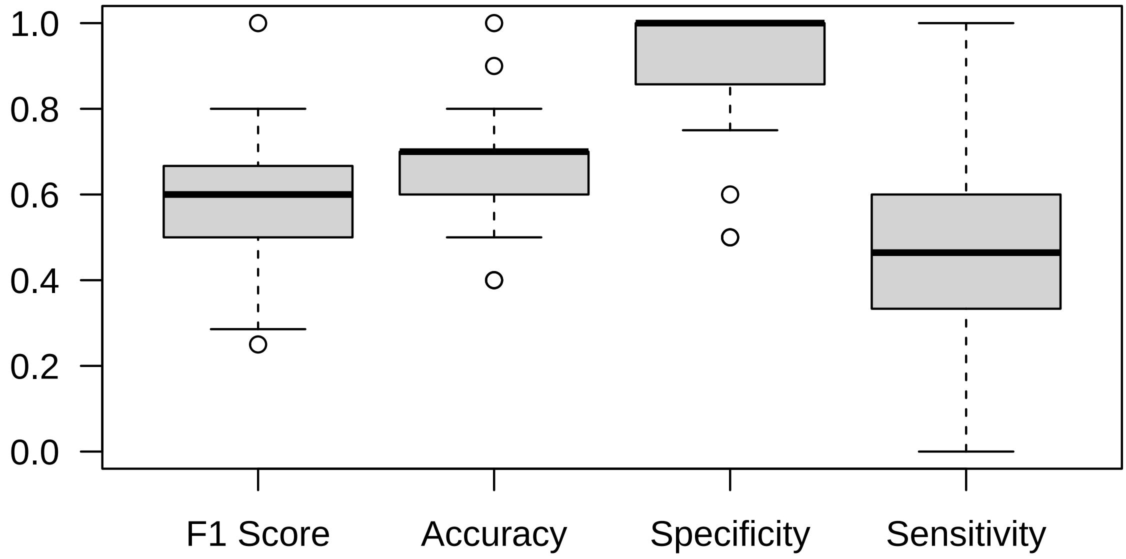

Figure S10: **B cells shared clones enable weak COVID-19 classification**
Samples were randomly split to train and validation groups. Shared clones were counted in the training group, and logistic regression was trained on tables summarizing the frequency of each clone in all training samples. Classifications were then made for the validation group. A Boxplot representing the F1 score, accuracy, specificity, and sensitivity of classifications of 50 random splits to train and validation groups is shown.
